# A comparative study of a novel absorbable cranial flap fixation system and Aesculap CranioFix

**DOI:** 10.1186/s41016-025-00406-6

**Published:** 2025-09-13

**Authors:** Chubei Teng, Hong Liang, Qi Yang, Yuan Fang, Zhihong Jian, Jianbai Yu, Gang Luo, Xiaoqin Han, Junjun Du, Siyi Wanggou, Xuejun Li

**Affiliations:** 1 Department of Neurosurgery, Xiangya Hospital, Central South University, Changsha, Hunan China; 2 Hunan International Scientific and Technological Cooperation Base of Brain Tumor Research, Xiangya Hospital, Central South University, Changsha, Hunan China; 3 Department of Clinical Research Management, West China Hospital, Sichuan University, Chengdu, Sichuan China; 4 Department of Neurosurgery, The First Affiliated Hospital of Hunan University of Traditional Chinese Medicine, Changsha, Hunan China; 5 Department of Neurosurgery, Renmin Hospital of Wuhan University, Wuhan, Hubei China; 6 MeiyiBoya Biomedical Technology Co., Ltd, Chengdu, Sichuan China

**Keywords:** Absorbable cranial flap fixation system, Multicenter clinical study, 3D reconstruction, Accelerated degradation test

## Abstract

**Background:**

Absorbable cranial flap fixation products, represented by Aesculap^®^ CranioFix absorbable clamp, are widely used in neurosurgery. However, the product has some shortcomings, as it is not entirely biodegradable, the lower disc’s angle cannot be adjusted, and there is a failure to readjust after fixation. To address these issues, MedArt Technology Co., Ltd. from China has come up with a high-purity PLLA combined with an innovative structural design to develop a novel cranial flap fixation system that is more convenient to operate, has a better resetting effect, and can be fully absorbed. This study aims to verify its safety and effectiveness through in vitro experiments and clinical trials.

**Methods:**

In this study, the absorbable cranial flap fixation system of MedArt was used as the experimental group, and the CranioFix absorbable clamp constituted the control group. The material properties and the changing trend of mechanical properties of the two groups were compared by accelerated degradation experiments in vitro. A multicenter, randomized, parallel, positive-controlled, non-inferiority clinical study was conducted with a 48-week follow-up. The shortening degree of the bone flap gap, qualified rate of bone flap displacement, changing trend of implant volume, and occurrence of postoperative adverse events were compared between the two groups.

**Results:**

The results of the in vitro accelerated degradation showed that in terms of the decrease in intrinsic viscosity, the control and experimental groups took 7 days and 14 days, respectively, to reach the test endpoint. For mechanical properties, the control group and experimental groups lost clinical safety fixation significance on the 3rd and 4th day after the degradation began, respectively. Regarding the release of degradation products, the control group showed a burst of lactic acid release during the first 3–7 days, while the experimental group released lactic acid slowly and constantly. In the clinical study, 90 patients were randomly enrolled, 87 of whom completed the operation, with an average age of 50. The 3D reconstruction of CT images showed that the bone flap gaps in both groups were less than 2 mm after surgery. The qualified rate of bone flap displacement in the experimental group was 100% after surgery. In contrast, in the control group, there was one unqualified case at 1 week after surgery and two unqualified cases at 6 weeks, 12 weeks, 24 weeks, and 48 weeks. The residual volume of the implant in the experimental group was closer to 50% (about 48.8%) 48 weeks after surgery, than in the control group (about 43.9%) 12 weeks after surgery. Regarding safety, only one possible device-related adverse event occurred in the control group, with an incidence rate of 2.22%, manifested as poor healing at the incision site.

**Conclusions:**

The study has verified that the experimental group had better stability, longer biodegradation time, and better mechanical properties than the control group. Moreover, the experimental group could significantly narrow the cranial flap gap, reduce the flap displacement, and promote skull healing after craniotomy. It shows a fairly reliable fixation effect and safety.

**Trial registration information:**

Registry: Chinese Clinical Trial Registry, registration number: ChiCTR2500099674, and date of registration: 27 March 2025 (https://www.chictr.org.cn/showproj.html?proj=261082).

**Supplementary Information:**

The online version contains supplementary material available at 10.1186/s41016-025-00406-6.

## Background

Globally, the number of neurosurgical operations is increasing with the rise of the incidence rate of craniocerebral diseases [1]. A craniotomy is a basic operation of neurosurgery. However, craniotomy often leads to complications such as skull resorption, skull defects, cerebrospinal fluid leakage, and meningoencephalocele due to poor bone flap healing [2]. Therefore, choosing appropriate materials and devices to fix the retracted bone flap stably is of great significance for maximizing the restoration of skull integrity, protecting brain tissue, reducing complications, and promoting rapid postoperative recovery of patients. The fixation method of the skull bone flap has undergone the evolution of suture, stainless steel wire, fixation plate, and fixation clamp [3]. The main limitation of sutures is their poor stability, which may lead to loosening and breakage, resulting in fixation failure. Although the stainless-steel wire can provide better stability and rigidity, the operation is cumbersome, and the uneven knot easily damages the scalp and causes exposure. Therefore, these two methods have been almost eliminated in clinical applications. Although the fixation plate exhibits good strength and toughness and is widely used in clinical practice, it is only fixed on one side. This limitation can lead to deformation under strong external forces, causing the bone flap to sink [4]. The fixation clamp uses a double-sided fixation method characterized by easy operation, fast tightening, firm clamping, and long-term implantation. It has become a commonly used device for skull bone flap fixation in craniotomy.

The fixation clamp achieves double-sided fixation through upper and lower discs, which has the following advantages:

1. Compared with the fixation plate, the operation of the fixation clamp is more convenient. Return the bone flap to the appropriate position, and after the upper and lower discs are firmly attached to the bone flap, the fixation can be completed.
2. Unlike fixation plates, fixation clamp does not require screws, thus avoiding skull penetration. It has no requirements for the thickness of the bone plate, making it more suitable for pediatric patients.
3. The connection strength between the upper and lower discs and the bite force between the disk and the skull determines the fixation force of double-sided clamping.

The skull mainly bears longitudinal pressure. Therefore, the mechanical stability of double-sided clamping is better than that of single-sided clamping, and it can ensure the smooth adhesion of the free skull bone flap and the barrier system at the edge of the bone window, which is more conducive to bone healing.

According to the biodegradability of the manufacturing material, the cranial flap fixation system is divided into nonabsorbable and absorbable products. Nonabsorbable products are usually made of titanium alloy or polyether ether ketone (PEEK). Titanium alloy is widely used in clinical practice due to its strong mechanical properties, wear resistance, and relative stability. However, there are problems such as CT or MRI imaging artifacts and slow release of harmful ions [5–7]. Therefore, titanium alloy is being gradually replaced by PEEK [8]. Although PEEK has good neurological safety, it is not as strong as metal materials, as it presents a long-term risk of breakage and wear and usually needs to be combined with other materials to improve performance [9, 10]. In addition, the screws used for fixation in permanent implant devices may move deep into the brain, and the tip may pierce the dura mater or even insert into the brain tissue, which may affect the growth of the skull in pediatric patients and cause developmental deformity [11–15]. As an emerging device for skull bone flap fixation, the absorbable fixation system offers several advantages, including biodegradability, toughness, non-paramagnetism, and magnetothermal effects. The core manufacturing materials are polylactic acid or polyglycolic acid, which have been utilized in clinical practice for many years [16, 17]. Currently, the absorbable products commonly used in clinics include the Aesculap^®^ CranioFix absorbable clamp, which is widely used in children’s craniotomy. However, the product still has problems such as incomplete absorption [18], inability to loosen and readjust after fixation, and inability to adjust the disc angle to fit the curvature of the skull [19], which affects not only the convenience of surgical operation but also the healing speed and effect due to uneven alignment of the bone flap.

In response to the above issues, MedArt used its self-developed high-purity poly-L-lactic acid (PLLA) to manufacture a fully absorbable cranial flap fixation system. This material has passed a complete set of biocompatibility tests, including in vitro cytotoxicity, sensitization, intradermal irritation response, pyrogen, hemolysis, acute systemic toxicity, subchronic systemic toxicity, genetic toxicity, and bone implantation. In addition, MedArt has innovatively designed the structure of an absorbable cranial flap fixation system, making implantation operations more convenient and beneficial for improving the healing effect of skull bone flaps.

To comprehensively evaluate the product’s performance from MedArt, this study used Aesculap® CranioFix absorbable clamp as the control product. It analyzed the mechanical properties, degradation time, and degradation products of the two products through in vitro accelerated degradation experiments. A multicenter, randomized, parallel, positive controlled, non-inferiority clinical trial was conducted to evaluate the effectiveness and safety.

## Methods

### Research object

The experimental group used an absorbable cranial flap fixation system (MedArt, Chengdu, China), and the control group used an Aesculap® CranioFix absorbable clamp (FF016/FF017, Aesculap, AG, Tuttlingen, Germany).

**Fig. 1:**
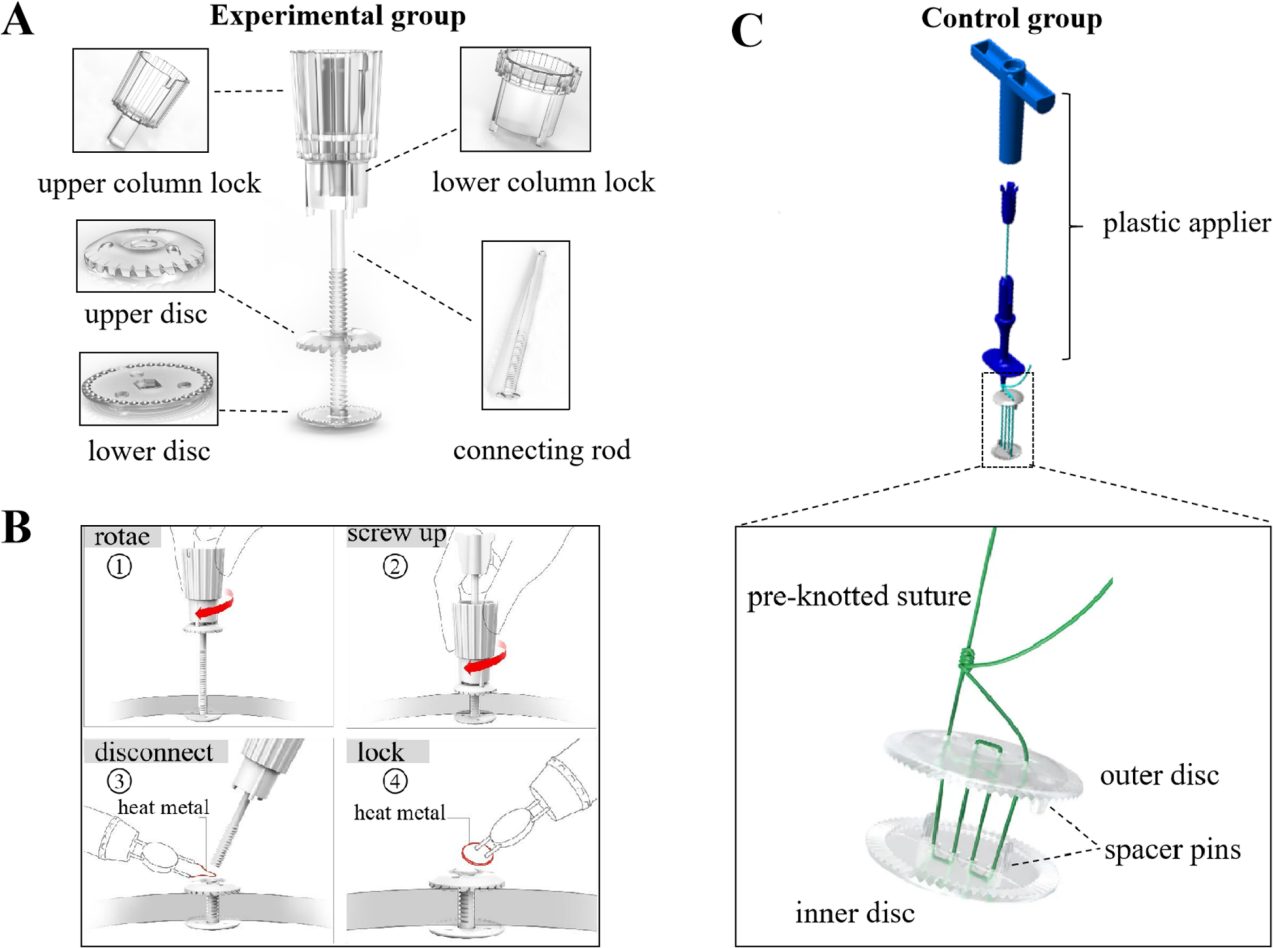
**A** Schematic diagram of absorbable cranial flap fixation system in the experimental group. The device mainly comprises an upper column lock, lower column lock, connecting rod, upper disc, and lower disc. **B** Schematic diagram of the fixed operating procedure of the experimental group device. **C** Schematic diagram of Aesculap® CranioFix absorbable clamp in the control group. The structure of the device mainly includes plastic applier, pre-knotted suture, outer disc, inner disc, and spacer pins

The structural characteristics of the products in the experimental group and the control group are shown in Fig. 1A and C. The fixation procedure of the absorbable cranial flap fixation system in the experimental group is shown in Fig. 1B. The characteristics and performance comparison of the two products are shown in Table 1.

**Table 1:** Comparison of the two bone flap fixation devices

Device	Experimental group	Control group
Materials	100% PLLA	Poly(L-lactide-co-D, L-lactide) 70:30, consisting of 70% L-lactic and 30% D-lactic acid
Structure	Column lock, connecting rod, upper disc, lower disc	Pre-knotted suture, outer disc, inner disc
Fixed mode	Clamp fixation	Clamp fixation
Features	✧ Completely biodegradable ✧ Preload is consistent, determined by the column lock ✧ Can be loosened and adjusted after fixation ✧ The diameter of the discs can be customized, with a minimum of 10 mm ✧ The fixing effect is determined by the connecting rod	✧ Incompletely biodegradable ✧ Preload is determined by the plastic applier ✧ Cannot be loosened and adjusted after fixation ✧ The diameter of the discs cannot be customized, with a fixed size of 11 mm ✧ The fixing effect is determined by the suture
In vitro accelerated degradation test	✧ Intrinsic viscosity: Drop below 0.1 dL/g on the 14th day ✧ Tripping force: Drop below 30 N on the 4th day ✧ Lactic acid release rate: Maintained in a steady state for the first 7 days and then released at a constant rate (15.42 mg/day) ✧ Crystallinity: First increase and then decrease	✧ Intrinsic viscosity: Drop below 0.1 dL/g on the 7th day ✧ Tripping force: Drop below 30 N on the 3rd day ✧ Lactic acid release rate: Sudden release occurred on the 7th day, with an average release rate of 40.09 mg/day ✧ Crystallinity: Non-crystalline
Postoperative follow-up (experimental group vs control group)	✧ Bone gap (mm): 1 week (1.61 vs 1.51), 12 weeks (1.00 vs 1.17), 24 weeks (0.79 vs 1.03), 48 weeks (0.58 vs 0.80), *P* < 0.05 ✧ Structural failure time: 24–48 weeks vs < 24 weeks ✧ The incidence of treatment-related adverse events (poor healing of incision site): 0% vs 2.22%	

### In vitroaccelerated degradation test

The product was immersed in a phosphate buffer solution at pH 7.4 to simulate the physiological state, and in vitro accelerated degradation test was carried out at 70 °C. The intrinsic viscosity, tripping force, crystallinity of the degraded sample, and lactic acid concentration in the degraded solution were measured. The dried samples at each time point were dissolved in chloroform, and the intrinsic viscosity was tested at 30 °C using an Ubbelohde viscometer. The enthalpy of the sample was measured by differential scanning calorimetry (DSC) to estimate the crystallinity. The tripping force was tested using a microcomputer-controlled electronic universal testing machine (CTM2050, Shanghai Xieqiang Instrument Technology Co., Ltd.). The lactic acid concentration of the degradation solution was determined by high-performance liquid chromatography (HPLC), and the same volume of buffer solution was added after each extraction.

### Clinical institutions and informed consent of participants

The Xiangya Hospital of Central South University led the clinical trials in collaboration with Renmin Hospital of Wuhan University, the First Hospital of Hunan University of Chinese Medicine, and West China Hospital of Sichuan University. All centers used uniformly designed clinical evaluation criteria to complete the effectiveness and safety observation of the patients. In addition, all participants involved in the study provided written informed consent.

### Inclusion/exclusion criteria

#### Inclusion criteria

1. Bone flap needs to be retracted and fixed after craniotomy,
2. 18 ≤ age < 70 years, (3) with simple intracranial space-occupying and nonmetastatic tumors such as aneurysms and meningiomas, (4) with craniocerebral trauma who needs fracture reduction and fixation and no risk of infection, and (5) the subjects or their family members voluntarily sign the “informed consent form” and can cooperate with postoperative follow-up, and cannot participate in other clinical trials within 9 months.

#### Exclusion criteria

(1) Need to remove the bone flap for decompression during craniotomy
Age < 18 years
Metastatic tumors or multiple intracranial lesions
Local scalp infection before surgery
Diabetes, hypertension, heart disease, and other underlying diseases
Lactic acid allergy
Mental illness
Pregnant or lactating and plan to become pregnant within 6 months
Participating in other clinical trials in the past 3 months
Unsuitable after comprehensive evaluation by the investigator.

**Fig. 2:**
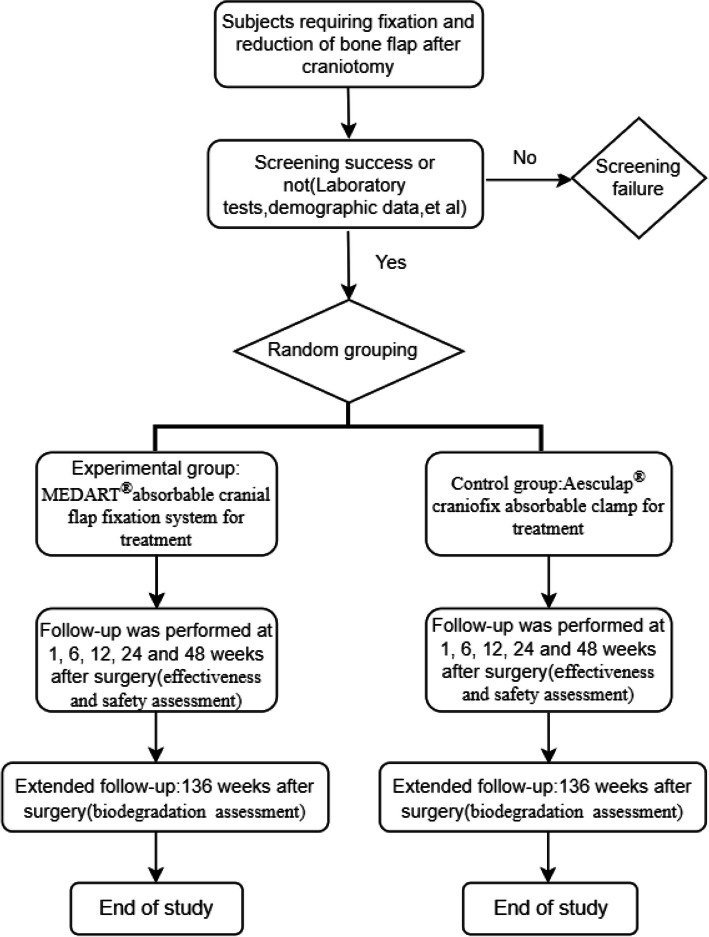
The flow chart of clinical trial

The flow chart depicting the clinical trial process is presented in Fig. 2.

### Protocol and evaluation criteria

This study adopted a multicenter, randomized, parallel, positive controlled trial method and selected non-inferiority evaluation to conduct statistical analysis on the main evaluation indicators of the trial. A central random system generated a random number table, and enrolled cases were randomly grouped according to the random number table. The clinical application of MedArt absorbable cranial flap fixation system was verified by comparing the effectiveness and safety of skull flap fixation in the two groups.

Effectiveness evaluation criteria are as follows: The primary outcome measure was the shortening distance of the bone gap at 12 and 24 weeks after surgery. The upper limit of the confidence interval of the mean difference in bone gap distance between the two groups is less than the non-inferiority margin, indicating that the experimental group is non-inferior to the control group. The secondary outcome measure was (1) the shortening distance of bone gap with computed tomography (CT) three-dimensional (3D) reconstruction at 48 weeks after surgery and (2) the qualified rate of bone flap displacement distance (< 2 mm was considered qualified) [20].

Safety evaluation criteria are as follows: (1) Treatment-related adverse events and (2) changes in clinical significance of various safety evaluation indicators before and after treatment.

### Data reconstruction

3D reconstruction of CT images was performed using the Materialise Mimics 17.0 software to obtain the bone gap distance data and set a uniform threshold range so that the reconstructed images could show the complete outline of implants in the two groups. The implant volume was obtained by dividing it from the surrounding tissue. CT image reconstruction and segmentation were completed under the guidance of experienced radiologists.

### Statistical analysis

SAS9.4 software was used for statistical analysis. Two-tailed tests were used for statistical tests. *P*-values ≤ 0.05 were considered statistically significant. The non-inferiority test was used for the primary effectiveness outcome measure, and the evaluation was performed using a two-tailed test with a test level of 0.05. Quantitative data were analyzed using Student’s *t*-test or Wilcoxon test, and qualitative data were analyzed using Pearson’s chi-squared test or Fisher’s exact test.

## Results

### In vitro accelerated degradation

**Fig. 3:**
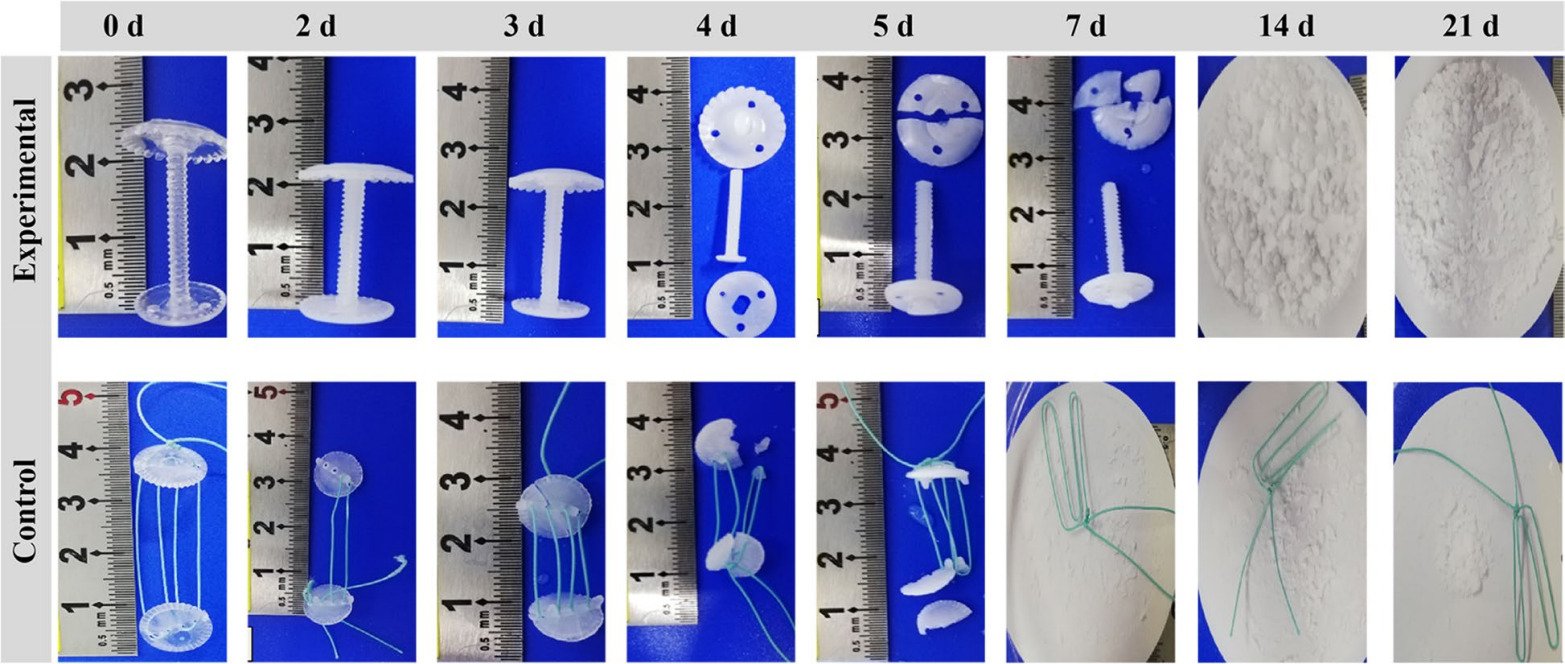
Photos of accelerated degradation in vitro at different times (0–21 days)

**Fig. 4:**
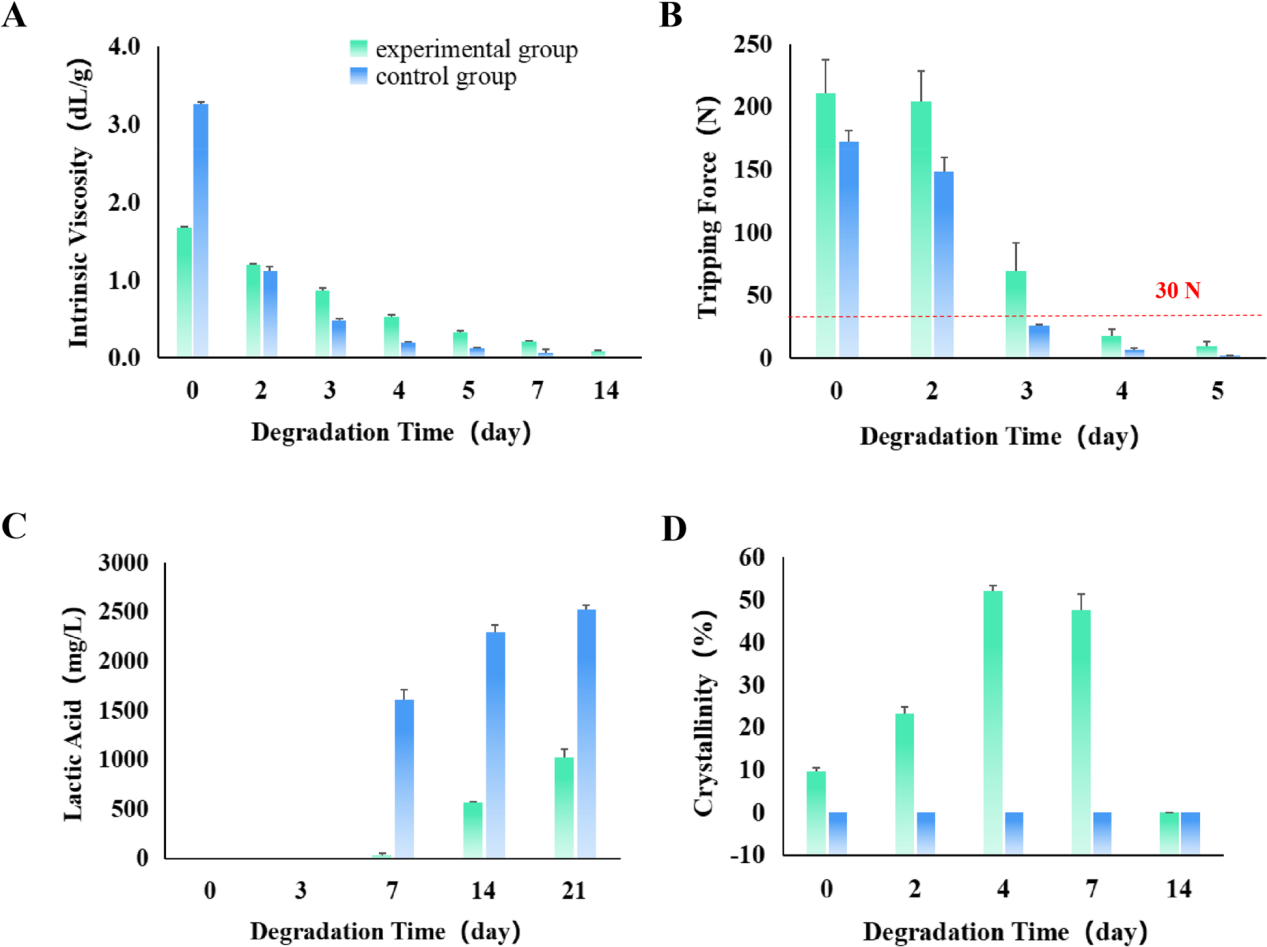
In vitro accelerated degradation test. **A** Intrinsic viscosity. **B** Tripping force. **C** Lactic acid concentration in the degradation solution. **D** Crystallinity. The green column represents the experimental group, and the blue column represents the control group. The red dashed line represents the minimum force with clinical safety fixation significance. Data represent mean ± SD (*n* = 3)

The photos of accelerated degradation in vitro at different times were recorded (Fig. 3). The results of in vitro accelerated degradation showed that the intrinsic viscosity change trends of the experimental group and the control group were consistent. The intrinsic viscosity of the experimental group decreased linearly in the first 5 days, and the decreasing rate was slower than that of the control group. The endpoint of the test was a reduction of intrinsic viscosity below 0.1 dL/g, which took 7 days in the control group and 14 days in the experimental group (Fig. 4A). In addition, under simulated physiological conditions, the changing trend of the tripping force of the two groups was consistent. The tripping force of the experimental group was better than that of the control group throughout the process. On the 3rd day after degradation, the tripping force of the two groups decreased sharply. The minimum force required for clinical safety fixation was 30 N, which was lost in the control group and the experimental group on the 3rd and 4th days after accelerated degradation (Fig. 4B). The degradation products of the two groups were lactic acid. The experimental group maintained a stable state for the first 7 days, releasing trace amounts of lactic acid. After 7 days, lactic acid was released at a constant rate of 15.42 mg/day until the endpoint. The control group showed a sudden release of lactic acid during the first 3–7 days, followed by a gradual decrease in the release rate over time, with an average release rate of 40.09 mg/day (Fig. 4C). In terms of crystallinity, the experimental group initially showed an increase, reaching a maximum value of (52.08 ± 1.25%) on the 4th day. However, the value decreased to 0% by the 14th day after accelerated degradation. In contrast, the control group showed no crystallinity from the onset (Fig. 4D).

The results of the in vitro accelerated degradation test showed that the degradation rate of the experimental group was slower than that of the control group. The time required for the intrinsic viscosity of the samples in the experimental group to drop to the test endpoint was about twice as long as that in the control group. In addition, when the release force dropped below the clinically safe fixed threshold, the experimental group also required longer than the control group. Regarding lactic acid release, the release rate of the experimental group was slower than that of the control group. The lactic acid release rate of the control group was more than twice that of the experimental group, and there was an evident burst release phenomenon in the control group. The crystallinity of the experimental group showed a trend of first increasing and then decreasing, which was consistent with the previously reported results [21]. As known already, polylactic acid’s glass transition temperature (*T*_g_) typically ranges from 50 to 60 °C [22]. When polylactic acid is maintained at *T*_g_ for an extended period, it can lead to conformational rearrangement [23]. Moreover, hydrolytic degradation mainly occurred inside the material rather than on its surface, and hydrolytic chain breakage occurred preferentially in the amorphous region, thus increasing the overall crystallinity of the polymer [24, 25]. The crystallinity of the control group was not detected from beginning to end, mainly because the crystallinity of amorphous polymers is usually very low or even without crystalline regions [26]. In summary, the results of the in vitro accelerated degradation test proved that the materials in the experimental group had better stability, showing longer degradation time and more robust mechanical properties.

### Clinical characteristics

The clinical trial commenced on September 4, 2017, and concluded on July 8, 2019. A total of 94 subjects were screened across all research centers, with 90 cases being randomly selected for enrollment, of which 87 underwent surgical procedures. The clinical characteristics of the two groups are shown in Table 2. The statistical analysis results showed no significant difference between the groups and were comparable. It should be emphasized that this study has excluded patients with basic diseases such as diabetes, hypertension, and heart disease.

**Table 2:** Clinical characteristics of the two groups

	Experimental group	Control group	*p*-value
Enrolled subjects	45	45	
Subjects of completed surgery	42	45	
Male (%)	18 (42.86)	16 (35.56)	> 0.05
Female (%)	24 (57.14)	29 (66.44)	> 0.05
Average age	50.07 ± 11.10	50.60 ± 12.62	> 0.05
History of treatment (%)	18 (42.86)	24 (53.33)	> 0.05

### Effectiveness assessment

**Fig. 5:**
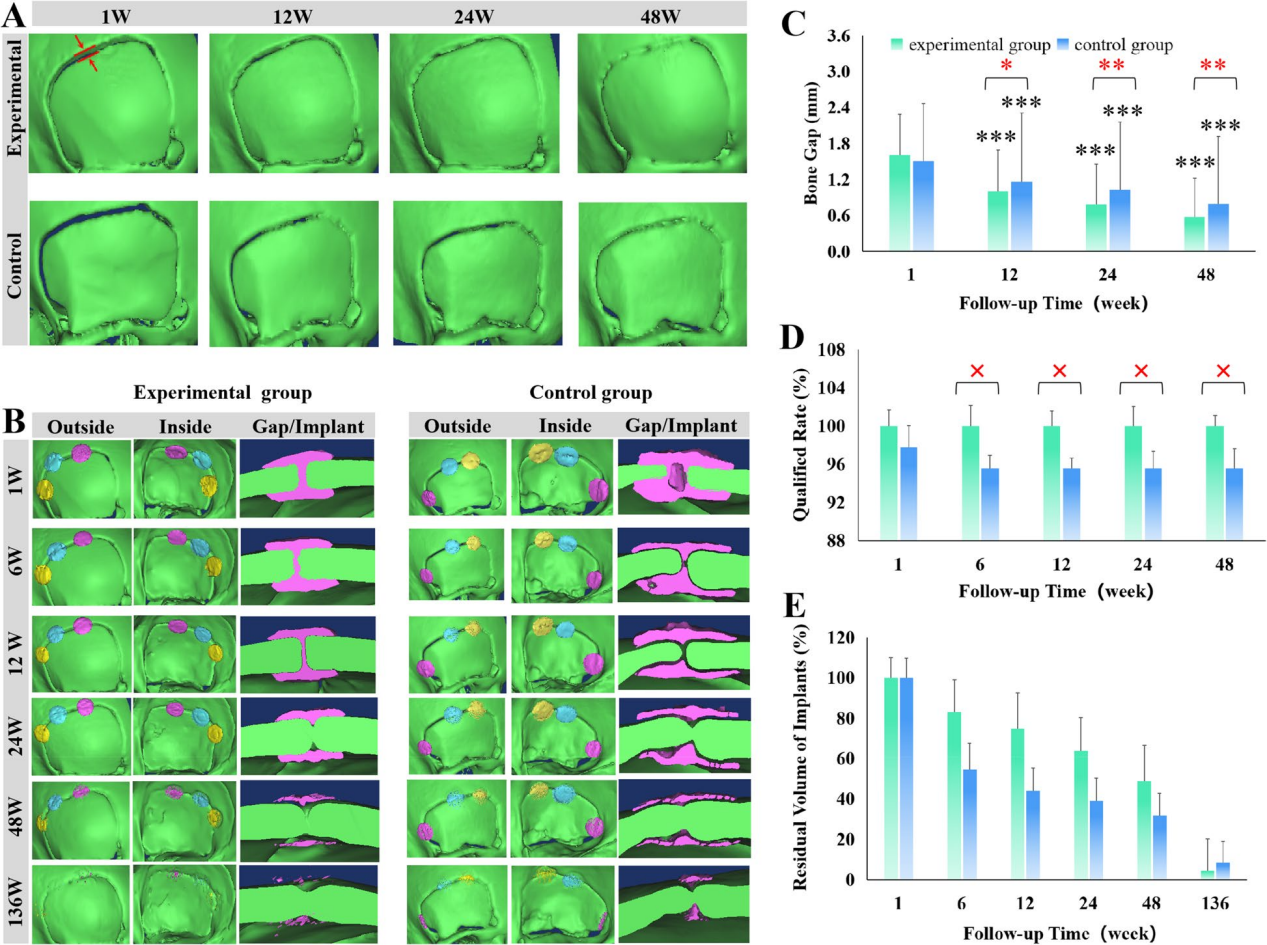
Postoperative effectiveness analysis. **A** 3D reconstruction of the CT images of the outside of the skull (1–48 weeks after surgery). The green part represents the skull and free bone flap, and the red arrow indicates the location of the bone gap. **B** The changes in bone gaps and implant volumes were analyzed using 3D reconstruction data (1–136 weeks after surgery). The purple, blue, and yellow circular parts represent implants in different locations. The purple rectangular part represents the volume of the implant. The gaps between the green rectangles are bone gaps. **C** Change trend of the bone gap from 1 to 48 weeks after surgery. **D** Qualified rate of postoperative bone flap displacement distance. **E** Residual volume change trend of implants. “*” represents *P* < 0.05, “**” represents *P* < 0.01, and “***” represents *P* < 0.001, all of which indicate that the difference is statistically significant. “ × ” represents *P* > 0.05, indicating that the difference is not statistically significant. Red marks indicate differences between groups, and black marks indicate differences within groups (compared with data from 1 week after surgery)

The bone gap distance was analyzed by 3D reconstruction of CT images (Fig. 5A and B). The bone gaps in the two groups gradually narrowed over time. Synostosis could be observed at 24 weeks and completed 48 weeks after surgery. The bone gap distance (mm) of the two groups (experimental group vs control group) at different time points was 1 week (1.61 vs 1.51), 12 weeks (1.00 vs 1.17), 24 weeks (0.79 vs 1.03), and 48 weeks (0.58 vs 0.80), respectively (Fig. 5C and Table 3). Taking the first week as the baseline, the differences within the groups were statistically significant after the *t*-test (*P* < 0.0001), and the differences between the groups were also statistically significant after analysis of covariance (*P* < 0.05).

**Table 3:** Postoperative bone gap distance (mm)

	Experimental group (*N* = 40)	Control group (*N* = 42)	*p*-value of difference between two groups
1 week	1.61 ± 0.68	1.51 ± 0.96	/
12 weeks	1.00 ± 0.69	1.17 ± 1.14	0.0279
24 weeks	0.79 ± 0.67	1.03 ± 1.13	0.0059
48 weeks	0.58 ± 0.64	0.80 ± 1.12	0.0058

The average displacement distance of the bone flap after surgery was less than 2 mm, which was considered qualified. At different time points after surgery, the qualified rate of bone flap displacement in the experimental group was 100%. However, in the control group, one case of nonconformance occurred 1 week after surgery, with a qualified rate of 97.78%. Two cases of nonconformance occurred at 6 weeks, 12 weeks, 24 weeks, and 48 weeks, respectively, with a qualified rate of 95.56% (Fig. 5D and Table 4).

**Table 4:** Qualified rate of postoperative bone flap displacement distance (%)

	Experimental group (*N* = 40)	Control group (*N* = 42)	*p*-value
1 week	100.00	97.78	/
6 weeks	100.00	95.56	0.3024
12 weeks	100.00	95.56	0.0776
24 weeks	100.00	95.56	0.4416
48 weeks	100.00	95.56	0.3477

In addition, the volume changes of the implants in the two groups were analyzed by 3D reconstruction of the CT images (Fig. 5B and E). The results showed that the volume of the implants in the two groups gradually decreased with the increase in degradation time. However, the volume changes of the implants in the experimental group were smaller than those in the control group at all time points. The experimental group implants were not significantly degraded until 24 weeks after surgery, with a residual volume of approximately 63.6%, and the residual volume was still close to 50% (approximately 48.8%) at 48 weeks. However, in the control group, significant degradation was observed in the 6th week after surgery, with the remaining volume of about 54.6% and less than 50% (about 43.9%) in the 12th week.

### Safety assessment

In this study, various blood indicators were tested (Table S1), including 4 blood routine indicators (RBC, WBC, HGB, PLT), 10 blood biochemical indicators (ALT, AST, TBIL, DBIL, ALP, GGT, BUN, CREAT, K, CA), and 2 coagulation function indicators (APTT, PT). There was no difference between the two groups in blood test results 1-week pre-surgery and 1-week post-surgery (*P* > 0.05).

The postoperative complications observed in this study mainly include infection and inflammation, epidural hematoma/effusion, subcutaneous hematoma/effusion, and poor local incision healing (Table 5). Infection and subcutaneous effusion were common postoperative complications. In this study, the infection symptoms of the subjects were mild, and the symptoms eventually disappeared after symptomatic treatment. No targeted treatment was carried out for subcutaneous effusion, and the symptoms eventually disappeared. Only one case of infection and poor healing of the local incision was observed in the control group. Since these symptoms disappeared after treatment, the researchers considered them “possibly related” to the device.

**Table 5:** Postoperative complications

Complications	Device	Follow-up time				
		1 week	6 weeks	12 weeks	24 weeks	48 weeks
Infection and inflammation	Experimental	11	0	1	0	0
	Control	11	0	0	0	0
Epidural hematoma/effusion	Experimental	0	1	1	1	0
	Control	3	0	0	0	0
Subcutaneous hematoma/effusion	Experimental	1	0	0	0	0
	Control	1	0	0	0	0
Poor healing of the local incision	Experimental	0	1	0	0	0
	Control	0	1	1	0	0

The frequency statistics of treatment-related adverse events (Table S2) and serious adverse events (Table S3) are shown in the supplementary materials. Overall, there were no device-related adverse events in the experimental group. One case of adverse event possibly related to the device occurred in the control group, with an incidence rate of 2.22%, manifested as poor healing at the incision site. No device defects that could lead to serious adverse events were observed in either group.

This study used CT 3D reconstruction technology to quantitatively analyze the changes in bone gaps to accurately evaluate the effect of skull healing [27, 28]. Moreover, the method could separate the implant from the surrounding tissue by adjusting the threshold range to analyze the implant’s biodegradation visually. In this study, the clinical effects of the two groups of products were compared in terms of bone healing, in vivo biodegradation, and complications.

The results of multicenter clinical trials showed that the bone gaps were less than 2 mm in two groups at different follow-up time points. Furthermore, the bone gaps in the experimental group were significantly smaller than those in the control group (*P* < 0.05). It was worth noting that significant biodegradation of the experimental group products was not observed until 24–48 weeks after surgery, while the control group products ultimately failed in structure within 24 weeks after implantation. The main reason for this result was the different crystallinity of the two materials. Previous studies have shown that amorphous materials degrade quickly due to their low crystallinity [29]. Bone healing was a complex process generally thought to take 12–24 weeks, and complete healing often took 48 weeks or even longer [30, 31]. Therefore, the structure failure time of the absorbable fixation device should be as close to the healing endpoint as possible to ensure its expected clinical effect. Another focus was safety. The results showed that no serious adverse events occurred in either group, and only one case of potentially device-related adverse event occurred in the control group.

In summary, compared with the control group, the experimental group showed a better bone healing effect, attributed to a combination of factors such as stronger fixation of the bone flap, longer biodegradation cycle, and fewer postoperative complications in the experimental group. These differences were directly related to the device’s structural design and manufacturing materials. The results of in vitro experiments and clinical trials showed that MedArt’s absorbable cranial flap fixation system has achieved better clinical effectiveness and safety than similar representative products through structural innovation and material optimization.

## Discussion

### Structural characteristics

Specifically, in terms of structural design, the control product consisted of two absorbable discs with slightly warped edges with gap pins and nonabsorbable sutures for connecting the upper and lower discs [18, 19]. The upper and lower discs clamp the bone flap to achieve firm fixation by tightening the suture with the clamp applicator. This method could not ensure the consistency of clamping force. Moreover, the position of the fixation device might need to be readjusted according to the actual situation. However, the control group product could not be loosened and adjusted after clamping, and the only option was to cut the suture and reinstall a new device, which affected surgical efficiency and led to unnecessary waste. In addition, although the gap pins in the upper and lower discs prevented horizontal displacement of the device, they also limited the angle of the discs, which affected the fit of the discs to the skull.

The experimental group product adopted the method of tightening bolts to fix the two clamping discs. The product’s structure mainly included five parts: the upper column lock, the lower column lock, the connecting rod, the upper disc, and the lower disc. The column lock drove the upper disc downward, and a locking structure formed between the connecting rod and the upper and lower discs fixed the bone flap.

The column lock adopted an innovative fixed-force column design to ensure consistent preload during fixing. The lower column lock and fastening hole on the upper disc were perfectly fitted to ensure a stable connection during the fixing process. In addition, before removing the column lock, if the fixing effect is unsatisfactory, the operator can loosen the upper disc by rotating the lower column lock in reverse, adjusting the device’ position, and tightening it again. Therefore, in practical clinical applications, the experimental group products did not have significant disadvantages in terms of operational steps and time consumption compared to the control group products.

It was worth mentioning that the connecting rod in the experimental group product adopted a flat and threaded design, which significantly improved the stability of the device. Moreover, the riveting structure formed by cutting and ironing the connecting rod with the upper disc could make the device more stable and prevent loosening. The unique flattened design made the rod suitable for narrower bone gaps, which was beneficial for healing bone flaps. Moreover, the flattened design could increase the contact area between the connecting rod and the edge of the bone window, which was beneficial for limiting the horizontal displacement of the device. The hanging platform structure at the end of the connecting rod separated it from the lower disc, thereby increasing the mobility of the lower disc and enabling the angle to be flexibly adjusted to fit perfectly with the skull’s inner surface.

The first anti-slip tooth on the upper disc increased the friction with the skull’s outer surface. The second anti-slip tooth on the lower disc was designed with raised dots, making the lower disc the thinnest among similar products, thereby reducing the peeling of the dura mater. Moreover, the hole on the lower disc for inserting the connecting rod was designed as a rectangle, which was conducive to limiting the movement of the lower disc and effectively avoiding damage to the dura mater caused by the movement of the lower disc during the tightening process. In addition, the drainage hole design in the lower disc could effectively avoid the formation of effusion and dead space in the fixed position, reducing the risk of re-craniotomy.

### Manufacturing materials

The upper and lower discs of the control group were made of a copolymer of poly(L-lactide-co-D, L-lactide) (PLDLLA, molar ratio L-lactide/D, L-lactide = 70/30), and the sutures used for fixation were nonbiodegradable polyester. As a result, the organism could not completely absorb the control group products, leading to a potential risk of long-term foreign body rejection. On the other hand, PLLA was a semicrystalline polymer, and PLDLLA was an amorphous polymer, resulting in slower degradation of PLLA and relatively faster degradation of PLDLLA [32]. Therefore, the control group product used PLDLLA probably to avoid the long-term implantation risk caused by slow degradation. However, studies have shown that the D-lactic acid produced by the degradation of poly(D-lactide) materials is harmful to the human body [33–35]. Therefore, all structures of the experimental group products were made of 100% PLLA material, which could not only be completely degraded but also has higher crystallinity, chemical stability, and resistance to enzymatic degradation compared with PLDLLA. Moreover, since the L-lactic acid produced by PLLA degradation was harmless to the human body, there was less long-term safety risk to the brain and nervous system [36]. In addition to crystallinity, the degradation rate of polymers was also related to molecular weight, with higher molecular weight resulting in a slower degradation rate. Therefore, the experimental group used high-purity PLLA materials with lower molecular weight, which not only avoided the harm of D-lactic acid but also solved the problem of slow degradation of high-molecular-weight PLLA while meeting the mechanical performance requirements for clinical applications.

### Subjects age

Compared to permanent fixation devices, absorbable fixation systems do not affect the growth and development of children’s skulls, making them more suitable for pediatric patients. However, from a safety perspective, this study only included participants aged 18 to 70 years old. Adults have better tolerance and can better handle and treat potential adverse events, thereby reducing risk. Therefore, in clinical trials that focus on verifying the effectiveness and safety of the product, we chose adult subjects.

## Conclusions

This study systematically evaluated the effectiveness and safety of a novel absorbable cranial flap fixation device through the in vitro accelerated degradation test and the multicenter clinical trials. In vitro studies have shown that compared with the control group, the experimental group had better stability, longer degradation time, and more robust mechanical properties. Clinical studies have shown that all evaluation indicators of the experimental group were non-inferior to those of the control group. Compared with the control group, the experimental group products had a more convenient operation, more stable fixation, and faster bone healing. In this study, the main clinical risks of the experimental group products were identified and controlled, and the residual risks were acceptable. The success of MedArt’s absorbable cranial flap fixation system was mainly due to the innovative design of the device structure and the optimization of the manufacturing materials. The results of this study were of great significance for promoting the innovation and development of absorbable cranial flap fixation devices.

## Supplementary Information

Additional file 1. Table S1 Hematological examination. Table S2 Statistics of treatment-related adverse events. Table S3 Statistics of serious adverse events

## Data Availability

Data cannot be shared publicly, as it pertains to the privacy and sensitive information of the participants. Data are available from the Medical Ethics Committee of Xiangya Hospital Central South University (contact via xyyyllwyh@126.com) for researchers who meet the criteria for access to confidential data.
