# Supplementary material for: A comparative study of a novel absorbable cranial flap fixation system and Aesculap CranioFix
Source: Chin Neurosurg J. 2025 Sep 13;11:18. doi: 10.1186/s41016-025-00406-6 (PMC12433596; doi:10.1186/s41016-025-00406-6)
Supplement: Supplementary file 1 — Additional file 1. Table S1 Hematological examination. Table S2 Statistics of treatment-related adverse events. Table S3 Statistics of serious adverse events [file 41016_2025_406_MOESM1_ESM.doc]

**Supplementary Information**

**A comparative study of a novel absorbable cranial flap fixation system and Aesculap Cranfix**

Chubei Teng^1,2^, Hong Liang^6^, Qi Yang^1,2^, Yuan Fang^3^, Zhihong Jian^5^, Jianbai Yu^4^, Gang Luo^4^, Xiaoqin Han^6^, Junjun Du^6^, Siyi Wanggou^1,2^*, Xuejun Li^1,2^*

^1^Department of Neurosurgery, Xiangya Hospital, Central South University, Changsha, Hunan, China

^2^Hunan International Scientific and Technological Cooperation Base of Brain Tumor Research, Xiangya Hospital, Central South University, Changsha, Hunan, China

^3^Department of Clinical Research Management, West China Hospital, Sichuan University, Chengdu, Sichuan, China

^4^Department of Neurosurgery, The First Affiliated Hospital of Hunan University of Traditional Chinese Medicine, Changsha, Hunan, China

^5^Department of Neurosurgery, Renmin Hospital of Wuhan University, Wuhan, Hubei, China

^6^MeiyiBoya Biomedical Technology Co., Ltd, Chengdu, Sichuan, China

* Corresponding author E-mail:

1. Siyi Wanggou: zokygwong@gmail.com

2. Xuejun Li: lxjneuro@csu.edu.cn

**Table S1** Hematological examination

| Index | 1 week before surgery | | | 1 week after surgery | | |
| --- | --- | --- | --- | --- | --- | --- |
|  | Experimental group | Control group | P value | Experimental group | Control group | P value |
| RBC | 4.46±0.49 | 4.51±0.46 | 0.6016 | 3.63±0.57 | 3.77±0.61 | 0.2892 |
| WBC | 7.51±3.16 | 7.34±4.18 | 0.8285 | 10.66±4.19 | 11.12±4.38 | 0.6211 |
| HGB | 132.86±17.16 | 132.84±15.47 | 0.9971 | 108.02±17.10 | 112.05±17.29 | 0.2871 |
| PLT | 216.38±62.51 | 211.89±68.16 | 0.7500 | 218.68±80.15 | 201.86±68.16 | 0.3640 |
| ALT | 20.66±10.32 | 23.89±16.07 | 0.2715 | 25.70±29.62 | 33.97±67.88 | 0.4753 |
| AST | 21.55±6.97 | 24.90±15.34 | 0.1976 | 21.26±15.96 | 37.39±87.14 | 0.2468 |
| TBIL | 9.92±4.78 | 10.51±5.20 | 0.5845 | 8.85±3.85 | 8.91±5.30 | 0.9476 |
| DBIL | 3.80±(1.83) | 3.91±(1.95) | 0.7806 | 3.51±(1.36) | 3.56±(2.17) | 0.8873 |
| ALP | 80.60±21.87 | 74.73±22.87 | 0.2439 | 71.68±21.18 | 68.78±22.38 | 0.5552 |
| GGT | 25.76±32.00 | 36.12±70.54 | 0.3982 | 32.89±30.94 | 34.27±41.46 | 0.8678 |
| BUN | 5.01±1.66 | 4.77±1.36 | 0.4606 | 4.08±1.67 | 3.94±1.19 | 0.6656 |
| CREAT | 72.40±22.31 | 66.19±16.37 | 0.1408 | 70.77±23.22 | 65.71±17.78 | 0.2682 |
| K | 4.02±0.43 | 3.97±0.37 | 0.5633 | 3.75±0.39 | 3.84±0.53 | 0.4147 |
| CA | 2.25±0.11 | 2.26±0.13 | 0.6294 | 2.12±0.12 | 2.15±0.14 | 0.3836 |
| APTT | 31.88±4.51 | 29.99±4.39 | 0.0516 | 31.44±4.37 | 29.54±4.68 | 0.0594 |
| PT | 11.94±1.35 | 11.93±1.30 | 0.9892 | 12.82±1.73 | 12.87±1.83 | 0.8949 |

**Table S2** Statistics of treatment-related adverse events

| Adverse events | Experimental group（N=42） | | | | Control group（N=45） | | |
| --- | --- | --- | --- | --- | --- | --- | --- |
|  | Frequency | Subjects | Incidence rate (%) | Frequency | | Subjects | incidence rate (%) |
| Various injuries, poisoning, and surgical complications (poor healing of local incisions) | 0 | 0 | 0.00 | | 1 | 1 | 2.22 |
| Total | 0 | 0 | 0.00 | | 1 | 1 | 2.22 |

**Table S3 Statistics of** **serious adverse events**

| Classification | Experimental group（N=42） | | | Control group（N=45） | | |
| --- | --- | --- | --- | --- | --- | --- |
|  | Frequency | Subjects | Incidence rate (%) | Frequency | Subjects | Incidence rate (%) |
| Total | 9 | 8 | 19.05 | 10 | 8 | 17.78 |
| Various neurological disorders | 3 | 2 | 4.76 | 3 | 3 | 6.67 |
| Epilepsy | 1 | 1 | 2.38 | 1 | 1 | 2.22 |
| Cerebrospinal fluid leakage | 0 | 0 | 0.00 | 1 | 1 | 2.22 |
| Cerebral edema | 0 | 0 | 0.00 | 1 | 1 | 2.22 |
| Hemiparesis | 1 | 1 | 2.38 | 0 | 0 | 0.00 |
| Speech disorders | 1 | 1 | 2.38 | 0 | 0 | 0.00 |
| Various injuries, poisoning, and surgical complications | 1 | 1 | 2.38 | 4 | 3 | 6.67 |
| Epidural hematoma | 0 | 0 | 0.00 | 2 | 2 | 4.44 |
| Cerebral contusion | 0 | 0 | 0.00 | 1 | 1 | 2.22 |
| Brain hernia | 0 | 0 | 0.00 | 1 | 1 | 2.22 |
| Poor healing of the incision site | 1 | 1 | 2.38 | 0 | 0 | 0.00 |
| Endocrine system diseases | 1 | 1 | 2.38 | 1 | 1 | 2.22 |
| Hypopituitarism | 1 | 1 | 2.38 | 0 | 0 | 0.00 |
| Diabetes insipidus | 0 | 0 | 0.00 | 1 | 1 | 2.22 |
| Gastrointestinal system diseases | 0 | 0 | 0.00 | 2 | 1 | 2.22 |
| Nausea | 0 | 0 | 0.00 | 1 | 1 | 2.22 |
| Vomiting | 0 | 0 | 0.00 | 1 | 1 | 2.22 |
| Liver and gallbladder diseases | 1 | 1 | 2.38 | 0 | 0 | 0.00 |
| Hepatic insufficiency | 1 | 1 | 2.38 | 0 | 0 | 0.00 |
| Infectious and infective diseases | 1 | 1 | 2.38 | 0 | 0 | 0.00 |
| Pulmonary infection | 1 | 1 | 2.38 | 0 | 0 | 0.00 |
| Benign, malignant, and tumors of unknown nature (including cystic and polypoid) | 1 | 1 | 2.38 | 0 | 0 | 0.00 |
| Sarcoma | 1 | 1 | 2.38 | 0 | 0 | 0.00 |
| Eye organ diseases | 1 | 1 | 2.38 | 0 | 0 | 0.00 |
| Parophthalmia | 1 | 1 | 2.38 | 0 | 0 | 0.00 |
